# Disruption of CTCF at the miR-125b1 locus in gynecological cancers

**DOI:** 10.1186/1471-2407-12-40

**Published:** 2012-01-25

**Authors:** Ernesto Soto-Reyes, Rodrigo González-Barrios, Fernanda Cisneros-Soberanis, Roberto Herrera-Goepfert, Víctor Pérez, David Cantú, Diddier Prada, Clementina Castro, Félix Recillas-Targa, Luis A Herrera

**Affiliations:** 1 Unidad de Investigación Biomédica en Cáncer, Instituto Nacional de Cancerología (INCan)-Instituto de Investigaciones Biomédicas, Universidad Nacional Autónoma de México (UNAM), México, DF, México; 2 Departamento de Patología, INCan, México, DF, México; 3 Instituto de Fisiología Celular, Departamento de Genética Molecular, UNAM, México, DF, México; 4 Instituto de Investigaciones Biomédicas, UNAM, P.O. Box 70-228, Ciudad Universitaria, 04510 México, DF, México

**Keywords:** CTCF, *miR-125b1*, Epigenetic, Cancer, Promoter, MicroRNA, Breast cancer

## Abstract

**Background:**

In cancer cells, transcriptional gene silencing has been associated with genetic and epigenetic defects. The disruption of DNA methylation patterns and covalent histone marks has been associated with cancer development. Until recently, microRNA (miRNA) gene silencing was not well understood. In particular, miR-125b1 has been suggested to be an miRNA with tumor suppressor activity, and it has been shown to be deregulated in various human cancers. In the present study, we evaluated the DNA methylation at the CpG island proximal to the transcription start site of *miR-125b1 *in cancer cell lines as well as in normal tissues and gynecological tumor samples. In addition, we analyzed the association of CTCF and covalent histone modifications at the *miR-125b1 *locus.

**Methods:**

To assess the DNA methylation status of the miR-125b1, genomic DNA was transformed with sodium bisulfite, and then PCR-amplified with modified primers and sequenced. The *miR-125b1 *gene expression was analyzed by qRT-PCR using U6 as a control for constitutive gene expression. CTCF repressive histone marks abundance was evaluated by chromatin immunoprecipitation assays.

**Results:**

The disruption of CTCF in breast cancer cells correlated with the incorporation of repressive histone marks such H3K9me3 and H3K27me3 as well as with aberrant DNA methylation patterns. To determine the effect of DNA methylation at the CpG island of *miR-125b1 *on the expression of this gene, we performed a qRT-PCR assay. We observed a significant reduction on the expression of *miR-125b1 *in cancer cells in comparison with controls, suggesting that DNA methylation at the CpG island might reduce *miR-125b1 *expression. These effects were observed in other gynecological cancers, including ovarian and cervical tumors.

**Conclusions:**

A reduction of *miR-125b1 *expression in cancers, correlated with methylation, repressive histone marks and loss of CTCF binding at the promoter region.

## Background

MicroRNAs (miRNAs) are a broad family of small non-coding RNAs and are involved in multiple cellular processes [1]. In mammals, miRNAs have been predicted to control the transcriptional activity of more than 60% of protein-encoding genes [2]. Therefore, miRNAs represent a new component associated with the regulation of gene expression [3]. The study by Sato and collaborators has gained importance because many miRNAs function as tumor suppressor genes and oncogenes and because their deregulation can lead to the development of cancer [3]. This phenomenon has been observed in lung, colon and breast cancer, among others. The deregulation of miRNAs is associated with both genetic and epigenetic defects [4,5]. Some miRNAs have been described to have an oncogenic function, such as *miR-17-92, miR-155 *and *miR-372-373*, and can influence cell proliferation, whereas others present tumor suppressor gene activity, e.g., as *miR-34, miR-26a *and *miR-125b *[2,6-8].

In particular, the expression of *miR-125b1 *was observed to be decreased in glioblastoma, prostate cancer, ovarian cancer and breast cancer. Interestingly, in vitro studies suggest that miR-125b1 targets HER2/neu and ESR1 genes, two genes important for the diagnosis and treatment of breast cancer [9,10]. One of the mechanisms associated with epigenetic silencing of miR-125b1 is DNA methylation [8]. Particularly, a hypermethylation of a CpG island located in proximity to the transcription initiation site was observed in cell lines and in tissue samples from patients with breast cancer [8,11].

It is now known that a multifunctional CCCTC-binding factor (CTCF) can serve as a barrier against the spread of DNA methylation and histone repressive marks over promoter regions of tumor suppressor genes. CTCF has been involved in many aspects of epigenetic regulation, such as X chromosome inactivation, genomic imprinting, regulation of non-coding transcripts and the structure and function of repeated elements [12,13].

CTCF has been involved in the epigenetic regulation of genes related to cell cycle control, such as BRCA1, Rb, p16 and p53 [14-18]. Recently, CTCF has also been shown to be involved in the regulation of miRNAs [19]. Therefore, CTCF may be able to protect against DNA methylation and the covalent incorporation of negative marks on histones in miRNAs with CpG islands [19].

The aim of the present study was to evaluate the DNA methylation at the CpG island proximal to the transcription start site of *miR-125b1 *in cancer cell lines as well as in normal tissues and gynecological tumor samples. In addition we analyzed the association of CTCF and covalent histone modifications at the *miR-125b1 *locus.

We found that the *miR-125b1 *CpG island is methylated in cancer cells. The disruption of CTCF combined with DNA methylation and the gain of repressive histone covalent marks, such as H3K9me3 and H3K27me3, result in the miRNA gene silencing.

## Methods

### Cell culture conditions

MCF-7, SK-BR-3, MCF10A and MDA-MB-231 cells were cultured in Dulbecco's modified Eagle's medium DMEM (Invitrogen) with 10% fetal bovine serum (Invitrogen) and 1% penicillin/streptomycin (Invitrogen, Carlsbad, CA, USA). All cells were grown in a humidified incubator at 37°C with 5% CO_2_.

### Tissue specimens and processing

All breast, ovarian and cervical cancer samples were obtained from the Instituto Nacional de Cancerología de México and classified according to the American Joint Committee on Cancer (AJCC) using the tumor-lymph node-metastasis (TNM) system. Diagnosis was given by trained pathologists from the institute based on WHO classification. Nine breast cancer samples, four ovarian cancer samples and three cervical cancer samples were obtained prospectively. These samples were obtained from untreated patients. As controls we analyzed three normal breast samples from mammoplasty surgeries, one normal ovarian sample from a hysterectomy indicated by cystocele, and one normal cervical sample from a healthy woman. Fresh samples were preserved at -20°C in RNAlater (Qiagen). Tissue sections were stained with H&E and were used only if they contained more than 70% cancer cells. This study was approved by the ethical committee of the Instituto Nacional de Cancerología (approval numbers 011/034/IBI and CB/727).

### Bisulfite sequencing and MS-PCR for the *miR-125b1 *promoter

DNA from tumor samples and cell lines were obtained by phenol/chloroform extraction. Two micrograms of genomic DNA was modified using the EZ methylation kit (ZYMO). For sequencing, modified DNA was subjected to PCR using oligonucleotides to amplify from the 573 to 303 positions upstream the transcription start site of *miR-125b1 *(Entrez Gene: 406911). We used the following primers for amplification (270 bp), F: 5'-TGGTGTTATAGGAGGTTGTG-3'; and R: 5'-ACCCAAATTTTTAAAACCATAA-3'. PCR products were purified with the DNA clean and concentrator kit (ZYMO Research). The products were cloned into the pGEM-T-Easy vector (Promega). At least sixth clones were selected randomly for DNA sequencing.

MS-PCR was performed with DNA treated with sodium bisulfite. The following primers were designed using MethPrimer software. For methylation analysis MSPmet125b1F 5'-TGGTGTATCGTTTTTTGTTTTC-3' and MSPmet125b1R 5'-ACCCATTCGAAACGAAAC, and for unmethylated analysis MSPunm125b1F 5'-ATTTGGTGTATTGTTTTTTGTTTTT and MSPunm125b1R 5'-CTCACCCATTCAAAACAAAAC. As a positive control, 1 μg of DNA from lymphocytes of a healthy donor was methylated in vitro (IVD) for eight hours using SssI methyltransferase (NEB, Beverly, MA).

### ChIP assay

Standard chromatin immunoprecipitation (ChIP) assays were performed as previously reported [18] with 4 μg of the following antibodies: H3K4me2 (07-030, Millipore); H3K9me2 (ab8898, Abcam); H3K27me3 (ab6002, Abcam), and CTCF (07-729, Millipore). As negative control we employed a normal rabbit IgG (sc-2027, Santa Cruz Biotechnology). Immunoprecipitated DNA was analyzed by PCR using specific primers to analyze the four genomic regions, including the *miR-125b1 *promoter. The 5' *p53 *gene promoter was used as positive control for CTCT's ChIP [18]; and for the H3K27me3 histone mark we analyzed the Myelin transcription factor 1 distal promoter (*MYT1*) [20]. Primer sequences used for ChIP assays are available by request.

### Quantitative real-time RT-PCR (qRT-PCR)

Total RNA was extracted with Trizol (Invitrogen). The miRNA levels were determined by qRT-PCR performed with cDNA generated from 10 ng of total RNA using the TaqMan^® ^miRNA Reverse Transcription Kit (Applied Biosystems). To evaluate the expression of the mature miR-125b1, we employed the TaqMan^® ^primer ID 002378 (Applied Biosystems). The relative amount of miR-125b1 was normalized against U6 snRNA (ID 001093), and the fold change was calculated by the 2-ΔΔCt method. The amplification and detection of specific products were performed with the ABI PRISM 7000 system (Applied Biosystems).

### Statistical analysis for qRT-PCR

Data from at least four independent experiments are expressed as the mean ± standard deviation. The differences between groups were analyzed using the Student's *t*-test comparing the cancer cell lines with a non-tumorigenic epithelial cell line (MCF10A), and breast tumor with normal breast samples. Data were considered significant if *p *< 0.001.

## Results

### DNA methylation profile of the *miR-125b1 *CpG island in breast cancer cell lines

**Figure 1:**
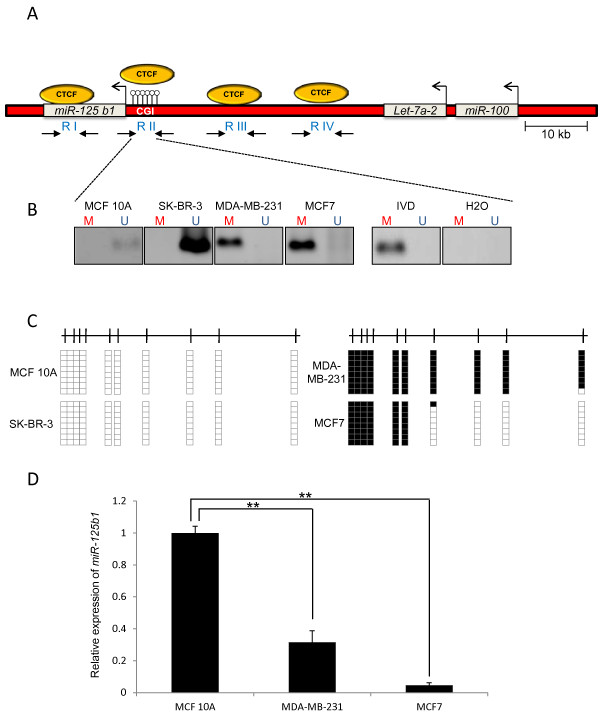
**Correlation between DNA methylation and expression status of *miR-125b1 *in breast cancer cell lines**. **A**- Schematic representation of the locus of *miR-125b1*. Circles represent the CTCF binding sites, open circles represent the CpG island (CGI) present upstream of *miR-125b1 *and arrows represent the transcription start site. The regions amplified by PCR for the chromatin immunoprecipitation (ChIP) assay are represented in arrows and named by regions as RI to RIV. **B**- Assessment of DNA methylation status using MS-PCR of miRNAs in the breast cancer cell lines SK-BR-3, MDA-MB-231 and MCF7. As a control for the technique, we assessed the methylation state of DNA from non-tumorigenic epithelial cell line MCF 10A. DNA from lymphocytes was methylated in vitro by SssI methyltransferase, and used as a positive methylated DNA. M represents methylated, and U represents non-methylated. **C**- Determination of the DNA methylation status using sodium bisulfite and sequencing in breast cancer cell lines. Black boxes represent methylated CpGs, and white boxes represent non-methylated CpGs. **D**- Expression analysis of *miR-125b1 *in breast cancer cell lines compared with MCF 10A.*P *< 0.001 Student's *t*-test.

As a first approach, we conducted an *in silico *study of the locus of *miR-125b1*. This miRNA is located on the long arm of chromosome 11 (11q24.1). *Let-7a2 *miRNAs and *miR-100 *are located upstream of this gene. In addition, there is a CpG island upstream from the transcription start site of *miR-125b1 *(Figure 1A). The DNA methylation status of the CpG island was assessed through sodium bisulfite conversion and MS-PCR in various transformed cell lines (Figure 1B). Using synthetic oligonucleotides modified for DNA methylation analysis, we determined that the CpG island associated with *miR-125b1 *is methylated in most breast tumor cell lines, with the exception of SK-BR-3 (Figure 1B, C). DNA from the non-transformed breast cell line MCF10A and DNA methylated in vitro by the enzyme Sss I (IVD) were used as controls (Figure 1B). These data suggest that DNA methylation of the *miR-125b1 *CpG island primarily occurs in transformed cell lines (i.e., MDA-MB-231 and MCF7). To confirm the DNA methylation status, we cloned and sequenced the product of sodium bisulfite conversion (Bs-seq) for the MDA-MB-231, MCF7, SK-BR-3 and MCF10A cell lines (Figure 1C). The results showed that these cell lines had different densities of DNA methylation, with partial methylation in MDA-MB-231 and in MCF7. In contrast, SK-BR-3 and the control, MCF10A, showed no methylation at the *miR-125b1 *promoter. These results suggest that DNA methylation of the *miR-125b1 *CpG island occurs mostly in transformed cells. In order to determine the effect of DNA methylation at the CpG island of *miR-125b1 *on the expression of this gene, we performed a qRT-PCR assay (Figure 1D). We observed a significant reduction on the expression of *miR-125b1 *in MCF7 and MDA-MB-231 in comparison with MCF10A, suggesting that DNA methylation at the CpG island might reduce *miR-125b1 *expression (Figure 1D). To confirm this hypothesis, we extended our study to samples from human gynecological tumors.

### DNA methylation profile at the *miR-125b1 *CpG island in primary gynecological tumor samples

**Figure 2:**
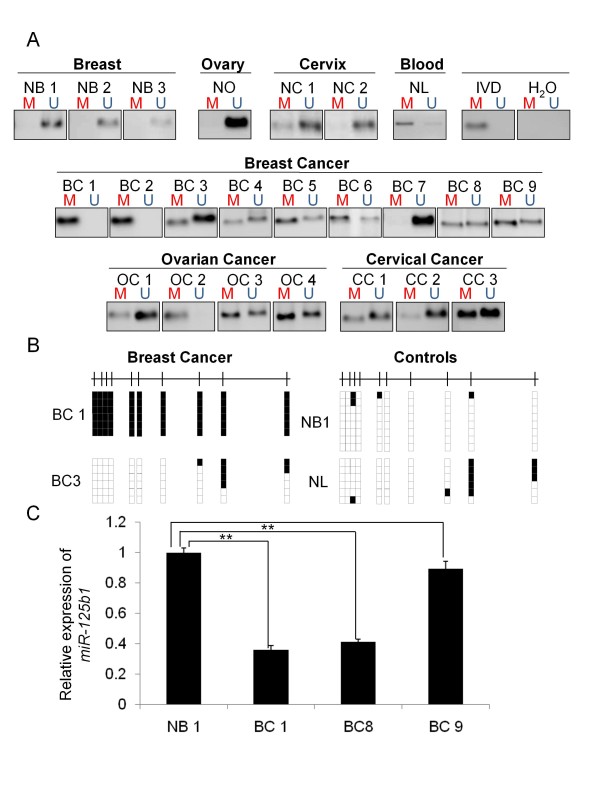
**Analysis of the DNA methylation status in normal tissue and gynecological tumor samples**. **A**- Analysis of DNA methylation by MS-PCR in several normal breast (NB), ovary (NO), cervix (NC) and peripheral blood lymphocytes (NL). Assessment of DNA methylation state in various gynecological primary tumors: Breast cancer (BC), ovarian cancer (OC) and cervical cancer (CC). **B**- Determination of the DNA methylation status using sodium bisulfite and sequencing in primary breast cancer samples and normal tissue samples. Black boxes represent methylated CpGs, and white boxes represent non-methylated CpGs. **C**- Expression analysis of miR-125b1 from primary breast cancer samples compared with normal breast tissue obtained during an aesthetic mammoplasty. **P < 0.001 Student's *t*-test.

Tumor samples were collected from breast (n = 9), ovarian (n = 4) and cervical tumors (n = 3) DNA was extracted from these samples and treated with sodium bisulfite. Subsequently, the DNA methylation status of the promoter of *miR-125b1 *was analyzed by MS-PCR. The methylation status for the breast tumor samples varied, 23% were totally methylated, 66% were partially methylated and only 11% showed no DNA methylation at the CpG island region of *miR-125b1*. As a control, we used normal breast tissue samples obtained from aesthetic mammoplasties. These control samples were analyzed by MS-PCR and Bs-seq, and no DNA methylation was detected in them (Figure 2A, B).

All together, these results suggest that DNA methylation at the CpG island of *miR-125b1 *occurs in tumors and could be involved in cancer development.

We then used this assay to analyze the methylation status in four ovarian cancer samples and three from cervical cancer. We observed DNA methylation of *miR-125b1 *in all samples from gynecological tumors except for one breast cancer sample, suggesting that this epigenetic modification is present only in transformed cells. Therefore, we evaluated the effect of the methylation at the CpG island on the expression of *miR-125b1 *in breast cancer samples by qRT-PCR. We found a significant decrease *in miR-125b1 *expression in breast cancer samples, BC1 and BC8, and a marginal reduction in BC9 sample compared with normal breast tissue. These results suggest that DNA methylation at the *miR-125b1 *CpG island decreases significantly the miRNA in human breast cancer samples. While the marginal reduction observed in sample BC9 could suggest that other epigenetic process might also be involved.

### Characterization of CTCF and histone marks across the *miR-125b1 *locus in normal and breast tumor samples

**Figure 3:**
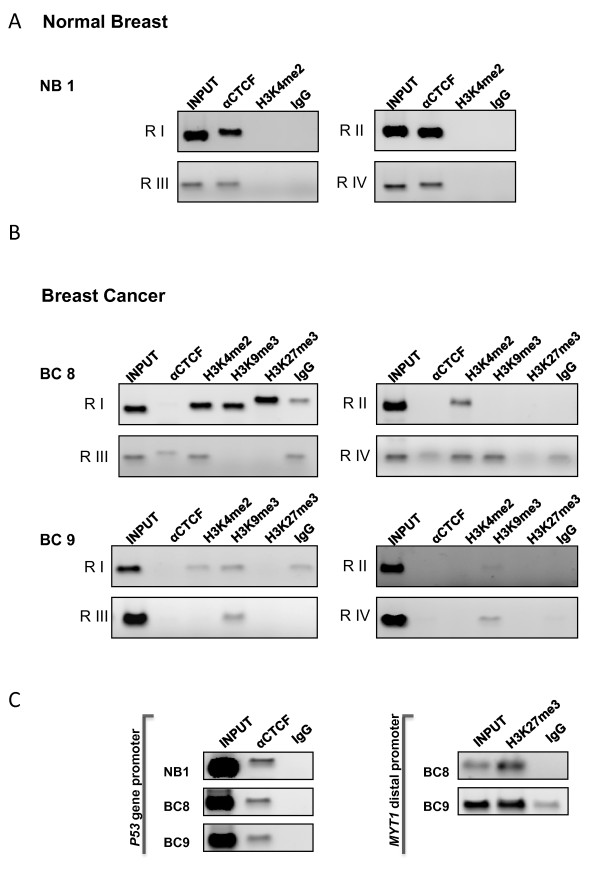
**In vivo CTCF binding analysis in *miR-125b1 *locus and the characterization of covalent histone marks in both normal cells and in primary breast tumors**. **A- **Chromatin immunoprecipitation (ChIP) of CTCF and the histone covalent marks analyzed at the human *miR-125b1 *locus in cells obtained from normal breast tissue (NB 1). **B**- Characterization of CTCF and histone covalent marks in two tumor samples tissue from different patients (BC8 and BC9), input amplification refers as the entire population of DNA. A non-specific IgG antibody was used as a control. **C- **Positive controls for the ChIP assay. For CTCF we performed a PCR of the *p53 *gene promoter region in normal breast (NB1) and two tumor samples (BC8 and BC9); for H3K27me3 we evaluated the abundance of the H3K27me3 histone mark at the Myelin transcription factor 1 distal promoter (*MYT1*) region in two tumor samples (BC8 and BC9).

Because CTCF has been proposed to protect genomic regions against DNA methylation, we decided to evaluate the presence of this factor using a ChIP assay in cells from normal breast tissue and from two breast cancer samples (Figure 2). This study was extended to four genomic regions of the locus of *miR-125b1 *(Figure 1A, RI-RIV). In addition, we analyzed the same regions for the presence of several covalent histone modifications such as H3K4me2, a histone mark associated with open chromatin, and the repressive histone marks H3K9me3 and H3K27me3 (Figure 3). An anti-IgG was included as negative control. For this assay, we used four different sets of primers, which amplified three regions located 3' and 5' from the *miR-125b1 *promoter, where it has been shown that CTCF binds in vivo [21]. In addition, we also used primers corresponding to the promoter region of the gene (Figure 1A). As a positive ChIP assay control for CTCF we evaluated the human *p53 *gene promoter in all our ChIPs, and for the H3K27me3 histone mark we analyzed the presences of this histone mark at the Myelin transcription factor 1 distal promoter (*MYT1*) region (Figure 3C).

In normal breast tissue, we observed CTCF enrichment in the four genomic regions, suggesting that this factor is probably linked to the chromatin structure and regulation of the *miR-125b1 *locus in cells from normal breast tissue (Figure 3A). Subsequently, we obtained chromatin from two breast tumors samples. Using the ChIP assay, we analyzed the covalent histone marks H3K4me2, H3K9me3 and H3K27me3. In the breast tumor cells we observed the loss of CTCF in all regions analyzed (Figure 3B). The gain of the negative histone mark H3K9me3 was observed in all regions in BC9 and in regions I and IV in BC8, while H3K27me3 was only observed in RI of BC8 (Figure 3B). These data strongly suggest that the absence of CTCF, together with DNA methylation and some histone repressive marks may contribute to the destabilization of chromatin permissive for transcription and the establishment of an aberrant repressive chromatin configuration of the *miR-125b1 *locus.

## Discussion

Cancer is primarily a disease that consists of the accumulation of genetic and epigenetic alterations. The epigenetic alterations can lead to the inactivation of tumor suppressor genes and the activation of oncogenes. Because some miRNAs target genes involved in cell cycle control, they have been associated with the functions of tumor suppressor genes and oncogenes [22]. Currently, there is experimental evidence that aberrant patterns of DNA methylation and repressive covalent histone modifications in the promoter regions of miRNAs associated with CpG islands can lead to epigenetic silencing [4]. Some studies have suggested that the loss of DNA methylation in the promoters of some miRNAs (e.g., *miR-21, miR-203 *and *miR-205*) in ovarian cancer results in their aberrant overexpression [23]. On the other hand, it has been shown that DNA hypermethylation in the promoters of *miR-124a *and *miR-127 *leads to their transcriptional silencing in colon cancer models and influences the expression of two oncogenes, such as BCL6 and CDK6 [4]. The epigenetic silencing of miRNAs has been frequently observed in early stages of breast cancer, including miR-9-1 and the miRNAs of the Let-7 family. Therefore, miRNAs could be good candidates for early tumor markers [24]. Furthermore, some miRNAs have been associated with invasion and metastasis in breast cancer [25]. One of the miRNAs that are deregulated in breast cancer is miR-125b1, and its expression is reduced in this type of cancer, suggesting its importance [10,26].

In the present study, we showed that methylation of DNA in a CpG island upstream of the transcription start site of *miR-125b1 *and the gain of repressive histone marks, such as H3K9me3 and H3K27me3, is a mechanism for the epigenetic silencing of this miRNA. We found that DNA methylation at the CpG island of *miR-125b1 *is present in the evaluated gynecological tumors. Our results suggest that this methylation negatively affects the miRNA gene expression, indicating that it could be in part responsible for the gene inactivation (Figures 1 and 2).

One of the most studied proteins in vertebrates is the nuclear factor CTCF [27]. This protein has the ability to delimit chromosomal borders, which affect the distinction of transcriptionally active regions from more compact regions in which transcription is repressed. It is currently accepted that CTCF can contribute to the establishment of chromatin domains. This organization promotes the creation of a local chromatin that facilitates communication between regulatory elements, such as promoters or enhancers [28]. Because CTCF has been related with protection against DNA methylation and repressive histone marks propagation, we investigated whether the presence of CTCF is associated with the transcription of *miR125-b1*. Interestingly, in normal breast cells, CTCF is distributed along the locus *miR-125b1*, whereas in primary breast tumors, we observed the absence of this factor. These data suggest that the absence of CTCF may be related to a gain of aberrant DNA methylation in the CpG island and a gain of repressive histone marks along the four genomic regions studied (Figure 3).

**Figure 4:**
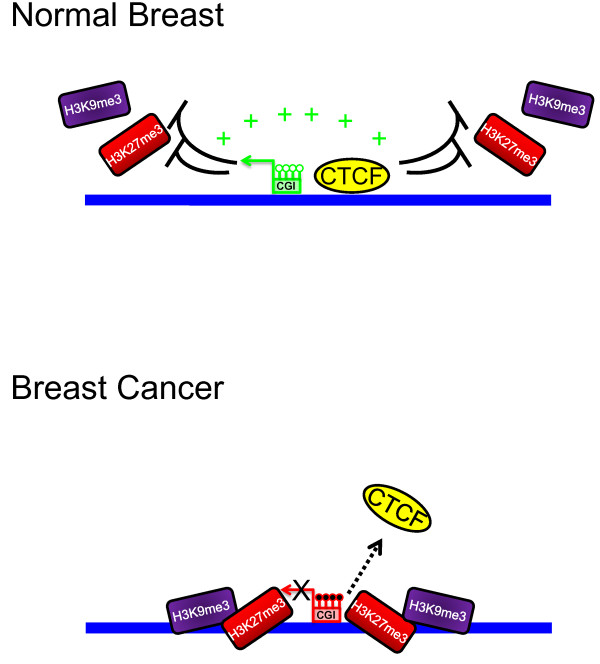
**Model of *miR-125b1 *gene silencing, in which the absence of the nuclear factor CTCF is associated with DNA methylation of the CpG island, and the enrichment of repressive histone marks**. In normal breast, CTCF might prevent the recruitment of the epigenetic silencing components, such as DNA methylation and repressive histone marks, also favors an open chromatin conformation (green color represent an open chromatin configuration). Meanwhile in breast cancer the loss of CTCF is associated with the CpG island (CGI) methylation and the gain of the repressive histone marks such as H3K9me3 and H3K27me3.

Therefore, we propose that the absence of CTCF along a locus may result in the loss of transcriptionally active borders, and together with DNA methylation and some histone repressive marks, such as H3K27me3, could promote the epigenetic silencing of the miRNA (Figure 4).

Some authors have referred to CTCF as the "glue" that connects the ends of domains, both intra- and inter-chromosomally, thus suggesting that deregulation of this factor leads to the aberrant silencing of multiple genes, including miRNAs [29]. These observations are consistent with published data that demonstrate that *miR-125b1 *can be silenced by DNA methylation, which may lead to a worse prognosis in cancer patients [8].

## Conclusions

Our results suggest that a reduction of *miR-125b1 *expression in cancers, is correlated with methylation, repressive histone marks and loss of CTCF binding at the promoter region.

## Competing interests

The authors declare that they have no competing interests.

## Authors' contributions

ESR and LAH designed methods and experiments, analyzed the results, and drafted the manuscript; RGB, performed the ChIP assays, and drafted the manuscript; FCS carried out the miRNA expression study; RHG and VP performed the histopathology of samples; DC enrolled and followed-up the patients; DP participated in the DNA methylation analysis; CC co-participated in the DNA methylation analysis; FRT analyzed and discussed the results, and drafted the manuscript. All authors have contributed to seen and approved the manuscript.

## Pre-publication history

The pre-publication history for this paper can be accessed here:

http://www.biomedcentral.com/1471-2407/12/40/prepub
